# Immunoliposome-based fluorometric patulin assay by using immunomagnetic nanoparticles

**DOI:** 10.1007/s00604-019-3973-9

**Published:** 2019-11-22

**Authors:** Xinjie Song, Danhua Wang, Myunghee Kim

**Affiliations:** Department of Food Science and Technology, Yeungnam University, Gyeongsan-si, Gyeongsangbuk-do 38541 Republic of Korea

**Keywords:** HPLC, Patulin, Rapid detection, Fluorescence intensity, Apple juice analysis

## Abstract

A fluorometric immunoassay is described for the determination of patulin, a highly toxic fungal metabolite. A rabbit anti-patulin-bovine serum albumin (BSA) IgG conjugate was prepared and used to compose immunoliposomes and immunomagnetic nanoparticles. The immunomagnetic nanoparticles are then added to the sample to form the patulin-antibody composites which can be magnetically separated. The immunoliposomes are then added to form a sandwich. After magnetic separation of the composites and adding n-octyl-β-D-glucopyranoside, the fluorophore sulforhodamine B (SRB) is released. Its fluorescence intensity was then measured at excitation/emission wavelengths of 550/585 nm. The immunoliposome-based immunomagnetic nanoparticle assay can detect 8 μg L^−1^ of patulin in apple juice without the need for extraction, separation, and purification. The detection limit falls within the European regulatory limit for infants and children’s products (10 μg L^−1^). The method is rapid, enviroment-friendly, and reliable.

**Electronic supplementary material:**

The online version of this article (10.1007/s00604-019-3973-9) contains supplementary material, which is available to authorized users.

**Graphical abstract:**
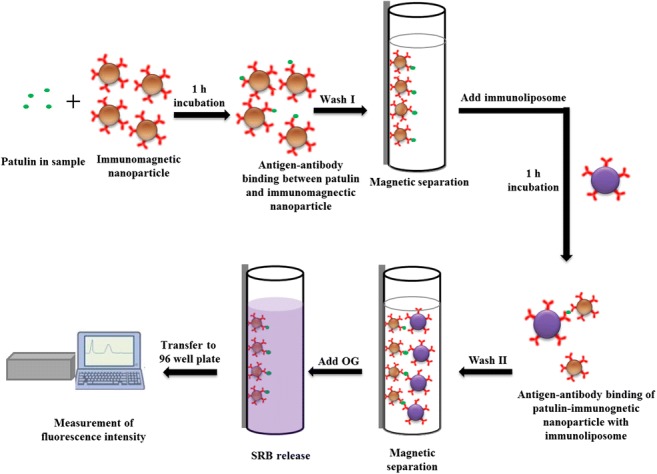
Schematic of the immunoassay.An immunoliposome-patulin-immunomagnetic nanoparticle sandwich complex is formed that can be separated from the sample by magnetic force. The fluorescence of sulforhodamine B carried inside of immunoliposome was measured to determine patulin. The method can detect 8 μg L^−1^ of patulin in apple juice.

## Introduction

Patulin is a toxic fungal metabolite produced by a number of fungal species, including *Aspergillus*, *Byssochlamys*, *Penicillium*, *Mucor*, and *Rhizopus*, which grow on foods, such as rotting apples and apple-derived products [1–3]. Several studies have demonstrated that patulin has genotoxic, immunotoxic, and neurotoxic effects in humans; therefore, patulin in food is considered an important risk factor for human health [4–6]. According to the European Union, the safe limits for patulin are 50 μg L^−1^ in apple juice and cider, 25 μg/kg in solid apples, and 10 μg L^−1^ in food products for infants and young children [7–9]. Thus, methods for the detection of patulin should be sensitive, selective, and reliable in order to monitor and control the patulin levels in foods.

Currently, the most widely used patulin detection method is chromatography in combination with other methods, including gas-liquid chromatography (GLC) with mass spectrometry, high-performance liquid chromatography (HPLC) with ultraviolet spectrometry, fluorescence, or mass spectrometry, and thin layer chromatography (TLC). For the chromatography methods, the concentration and separation processes are usually performed with organic solvents, which are environmentally deleterious [10]. Lately, several methods combined with surface plasmon resonance, fluorescent resonant energy transfer, and molecularly imprinted polymers have been reported for the detection of patulin [11–14]. Nanoparticles were applied in the detection of patulin to improve the methods (Table 1). Ma et al. reported a fluorometric aptasensor which used magnetic graphene oxide for patulin detection. The detection limit of the method was 0.28 μg L^−1^ with a linear range of 0.5 μg L^−1^ to 30 μg L^−1^ [15]. He and Dong reported an aptamer-based assay for patulin detection [16]. In this work, ZnO nanorods composed with chitosan modified gold electrode had good loading properties of aptamers. After the patulin reacted with aptamer on the gold electrode, the change in current of the electrode represented the patulin in the tested sample. The modified gold electrode has a narrow linear response of 0.5 μg L^−1^ to 50 μg L^−1^ and a detection limit of 0.27 ng L^−1^. Xu et al. reported a voltammetric method developed with Ag nanoparticle/Zn-based metal organic framework (MOF) nanocomposite [17]. The method has a detection limit of 4.64 μg L^−1^ with a wide linear range of 1.55 μg L^−1^ to 1.55 × 10^5^ μg L^−1^. Bagheri et al. also used mimetic Ag nanoparticle/Zn-based MOF nanocomposite to develop a fluorescence detection system for patulin [12]. The detection limit of the method was 9.24 μg L^−1^.

**Table 1:** Nanoparticles used in patulin detection method developed recently

Nanoparticle used	Method applied	Limit of detection	Linear range	References
Magnetized graphene oxide	Fluorescence resonanceenergy transfer	0.28 μg L^−1^	0.5 μg L^−1^ to 30 μg L^−1^	Ma et al., 2018 [15]
Au nanoparticle, ZnO nanorods	Voltammetric	0.27 ng L^−1^	0.5 μg L^−1^ to50 μg L^−1^	He and Dong, 2018 [16]
Ag nanoparticle/Zn-based MOF nanocomposite	Fluorometric analysis	9.24 μg L^−1^	15.4 μg L^−1^ to 1.54 mg L^−1^	Bagheri et al., 2018 [12]
Au nanoparticles, black phosphorus nanosheets	Voltammetric	4.64 μg L^−1^	1.55 μg L^−1^- to 1.55 × 10^5^ μg L^−1^	Xu et al., 2019 [17]
Magnetic nanoparticle, liposome	Fluorescence intensity	8.01 μg L^−1^	0–150 μg L^−1^	This work

Among the various nanoparticles, magnetic nanoparticles have great potential for application in rapid and sensitive methods due to their unique properties. Nanoparticles have large surface area per volume of the substance and strong magnetic response [18]. It makes the magnetic nanoparticles easy to modify and can be used in rapid separation and detection [19]. Magnetic nanoparticles are widely used in food safety to detect harmful substances, such as food-borne bacteria, toxins, and food additives [15, 18, 20]. Haghighi et al. (2019) used antibody-conjugated magnetic nanoparticles for highly efficient detection of HER2-expressing cancer cells in blood [21]. In other reports, antibody-conjugated magnetic nanoparticles have been developed for rapid separation and concentration methods for the detection of bacteria [22, 23]. Immunomagnetic nanoparticles are highly specific to the target due to the specificity of their antibody component. Their magnetic property enables effective and rapid separation. Using immunomagnetic nanoparticle to separate and concentrate the patulin is environment friendly; no need of using environmentally deleterious organic solvents. Similarly, liposomes are hollow spheres ranging from 20 nm to 10^4^ nm in diameter and enclosed by lipids [24]. Liposomes have large surface areas and relatively large encapsulation volumes, which make them excellent signal transducers [25]. Antibody-modified liposomes have been used in the detection of bacteria and viruses [23, 26]. Therefore, using immunomagnetic nanoparticles and immunoliposomes is an excellent strategy to develop a rapid detection method for patulin.

In this study, we synthesized a rabbit anti-patulin-bovine serum albumin (BSA)-immunoglobulin G (IgG)-modified immunoliposome, which encapsulated the fluorophore sulforhodamine B (SRB) as signal transducer. A magnetic iron nanoparticle, also surface modified with rabbit anti-patulin-BSA IgG, was used to capture the resultant patulin-immunoliposome composite. The fluorescence intensity of the immunoliposome-encapsulated SRB may be analyzed at an excitation wavelength of 550 nm and an emission wavelength of 585 nm using a fluorescence detector. The immunoliposome-based fluorometric patulin assay is rapid and environment-friendly for patulin detection.

## Experimental

### Reagents

The reagents 1,2-dipalmitoyl-sn-glycero-3-phosphocholine (DPPC), 1,2-dipalmitoyl-sn-glycero-3-phosphoethanolamine (DPPE), and 1,2-dipalmitoyl-sn-glycero-3-(phosphor-rac-[1-glycerol]) (DPPG) were purchased from Avanti Polar Lipids (Alabaster, AL, USA). Aditionally, acetic acid, acetonitrile, ammonium sulfate, caprylic acid, ethyl acetate, ethylenediaminetetraacetic acid (EDTA), dimethyl sulfoxide (DMSO), ethymaleimide, patulin, patulin-BSA, sodium sulfate, tris(hydroxymethyl)aminomethane (Tris), 4-(2-hydroxyethyl)-1-piperazineethanesulfonic acid (HEPES), methanol, n-octyl-β-D-glucopyranoside (OG), commercial rabbit IgG, protein markers, triethylamine, Tween 20, sucrose, chloroform, cholesterol, potassium phosphate dibasic, potassium phosphate monobasic, Sepharose CL-4B, sodium acetate, sodium azide, sodium carbonate, sodium chloride, sodium dodecyl sulfate (SDS), Freund’s complete adjuvant, Freund’s incomplete adjuvant, and polyacrylamide were purchased from MilliporeSigma (St. Louis, MO, USA). N-succinimidyl-s-acetylthioacetate (SATA), SRB, hydroxylamine hydrochloride, and N-(k-maleimidoundecanoyloxy) sulfosuccinimide (sulfo-KMUS) were purchased from Pierce Chemical (Rockford, MD, USA). Carboxyl magnetic iron oxide nanoparticle conjugation kits containing 1-ethyl-3-(3-dimethylaminopropyl)-carbodiimide/N-hydroxysuccinimide (EDAC/NHS) were purchased from Ocean Nano Tech (San Diego, CA, USA). Dialysis membranes (molecular weight cutoff, 12 to 14 kD) were purchased from Spectrum Laboratories (Rancho Dominguez, CA, USA). A magnetic separator was purchased from Dynal Biotech (Lake Success, NY, USA). Polycarbonate filters (0.4 μm and 0.8 μm pore sizes) and syringe filters were purchased from Whatman PLC (Maidstone, UK). Ninety-six-well microtiter plates were purchased from SPL Life Sciences (Pocheon, Gyeonggi-do, Republic of Korea). Patulin-free apple juice was purchased from a local market (Gyeongsan, Gyeongsangbuk-do, Republic of Korea).

### Immunogen preparation and immunization

Patulin-BSA was used as an immunogen in New Zealand white rabbits purchased from Samtako (Osan, Gyeonggi-do, Republic of Korea). The animals were treated according to the protocol and standards of the Canadian Council on Animal Care Guidelines. The prepared immunogen was mixed with an equal volume of Freund’s complete or incomplete adjuvants to immunize the rabbits. The antigen was injected to the back of rabbits at four sites with 0.25 mL/site. Blood samples were taken at the 18 week after first injection.

### IgG purification and characterization

Blood samples obtained from the immunized rabbits were centrifuged for 30 min at 10,000×*g* and 4 °C to separate the antiserum. IgG was purified from the antiserum using caprylic acid and ammonium sulfate precipitation [27]. Purification details are provided in the electronic supporting material (ESM). The purity of the rabbit anti-patulin-BSA IgG was confirmed by SDS-polyacrylamide gel electrophoresis (SDS-PAGE). The titer and specificity of the produced antibody were confirmed by indirect non-competitive enzyme-linked immunosorbent assay (INC-ELISA). A comparative analysis of the interaction of the antibody with ovalbumin, skim milk, and BSA was conducted using direct ELISA as shown in Fig. S3.

### Liposome and immunoliposome preparation

DPPE, DPPC, DPPG, cholesterol, and SRB were used to make a fluorescent dye-encapsulated liposome [27]. Preparation details are provided in the ESM. The rabbit anti-patulin-BSA immunoliposome was stored at 4 °C in the dark until further use.

### Immunomagnetic nanoparticle preparation

The anti-patulin-BSA IgG was conjugated to the carboxyl magnetic iron oxide nanoparticles according to the instructions on the conjugation kit and the method used by Shukla et al. [22]. Preparation details are furnished in the ESM. The immunomagnetic nanoparticles were resuspended in 1 mL of wash/storage buffer and kept at 4 °C before use. The average particle size before and after conjugation were measured using a Malvern Nano ZS particle size analyzer (Malvern, Worcestershire, UK) to confirm the conjugation.

### Liposome, immunoliposome, and immunomagnetic nanoparticle characterization

The sizes of the liposomes, immunoliposomes, magnetic nanoparticles, and immunomagnetic nanoparticles were determined using a Nano ZS particle size analyzer. We assumed that the concentration of the SRB encapsulated in the liposomes is equal to that of the original SRB solution. Therefore, the amount of SRB encapsulated in a single liposome can be calculated using the inner volume of the liposome particle. The concentration of the liposomes was calculated by dividing the total amount of SRB in the liposome solution by the amount of SRB inside a single liposome, resulting in particles/mL of liposome [27].

### Assay design

**Fig. 1:**
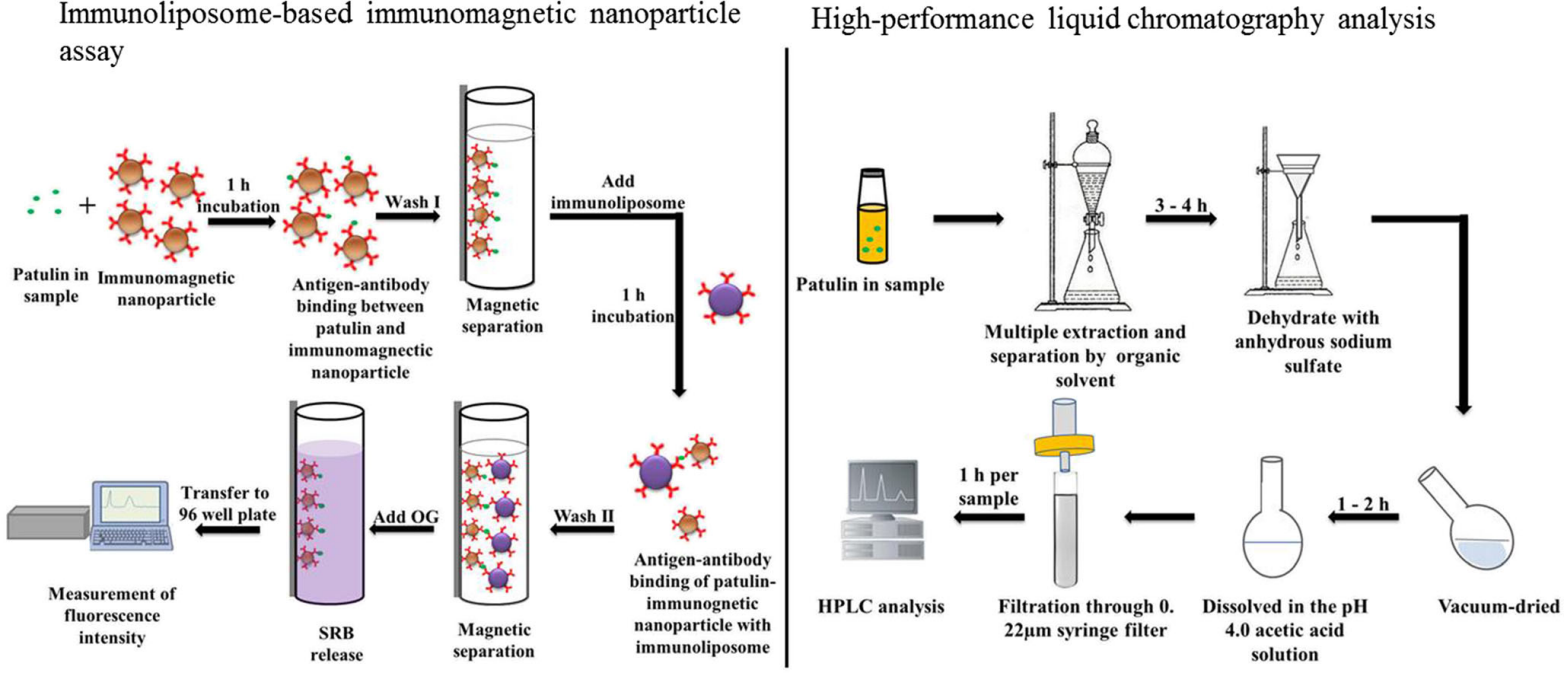
A systemic stepwise procedure of immunoliposome-based fluorometric patulin assay and high-performance liquid chromatography analysis

The stock solution of the immunoliposomes was diluted in the appropriate ratio (1:10) with 0.01 M TBS containing 0.04 M sucrose. The approach for the development of an immunoliposome-based immunomagnetic nanoparticle assay is shown in Fig. 1. First, 1 mL of diluted patulin or contaminated sample solution was put into a test tube, mixed with 20 μL of immunomagnetic nanoparticles, and incubated at room temperature for 1 h under constant shaking at 70 rpm. The tube was then inserted into the magnetic separator to separate the immunomagnetic nanoparticles bound with patulin. The patulin-immunomagnetic nanoparticle composites were washed with 1 mL of 0.01 M PBS containing 0.05% Tween 20. Next, 200 μL of the immunoliposome solution was added to the patulin-immunomagnetic nanoparticle composites and incubated at room temperature in the dark for 1 h. The immunoliposomes bound to the patulin-immunomagnetic nanoparticle composites were separated by a magnetic separator and then lysed with 260 μL of 30 mM OG. Finally, 200 μL of the lysed solution was transferred to a 96-well microtiter plate to measure the fluorescence intensity at an excitation wavelength of 550 nm and an emission wavelength of 585 nm using a fluorescence detector (Infinite M200, Tecan; Mannedorf, Switzerland). Various concentrations of patulin solution dissolved in 0.01 M PBS were analyzed to determine the detection limit.

### Fluorometric determination of patulin

This study aimed to develop a rapid, simple, and sensitive method for the detection of patulin in apple juice. Therefore, apple juice samples were spiked with different concentrations of patulin and analyzed using the optimized immunoliposome-based immunomagnetic nanoparticle assay. The limit of detection of the assay was determined based on the linearity function of the data and constant standard deviation of the responses to the calibrated standard and samples.

### Specificity of the assay

To confirm the specificity of the immunoliposome-based immunomagnetic nanoparticle assay, ochratoxin A was tested for comparison. Ochratoxin A was spiked into apple juice at concentrations of 10 μg L^−1^, 50 μg L^−1^, 100 μg L^−1^, 200 μg L^−1^, 500 μg L^−1^, 800 μg L^−1^, and 1000 μg L^−1^ in 0.01 M PBS and tested using the assay. To confirm the effects of BSA on the assay, different BSA concentrations were also spiked into apple juice and tested.

### Determination of patulin using HPLC

To confirm the efficacy of the immunoliposome-based fluorometric assay, a comparative analysis was demonstrated with a traditional standard method HPLC (Fig. 1). For HPLC analysis, patulin was extracted from apple juice samples artificially contaminated with 0 μg L^−1^, 10 μg L^−1^, 50 μg L^−1^, 100 μg L^−1^, 150 μg L^−1^, 200 μg L^−1^, 300 μg L^−1^, 400 μg L^−1^, and 500 μg L^−1^ patulin using ethyl acetate. The ethyl acetate fraction was treated with 0.5% sodium carbonate and passed through anhydrous sodium sulfate using a Buchner funnel [28]. The filtrate was vacuum-dried and dissolved in acetic acid solution (pH 4.0), then filtered using a 0.22 μm syringe filter and processed for HPLC analysis, which was carried out on an UltiMate 3000 HPLC (Thermo Scientific; Waltham, MA, USA) equipped with a UV detector. Briefly, a 20 μL sample was injected into a C18 column (5 μm pore size, 4.6 mm internal diameter × 250 mm length) (Waters; Milford, MA, USA) and eluted using 5% acetonitrile (*v*/v) at a constant flow rate of 0.5 mL/min. Patulin was detected at 276 nm and quantified by comparing the signal from samples with the signal from known concentrations of patulin.

## Results and discussion

### Antibody characterization

Details of antibody characterization are presented in the ESM.

### Liposome, immunoliposome, and immunomagnetic nanoparticle characterization

Details of the process are presented in the ESM.

### Determination of patulin using the optimized assay

**Fig. 2:**
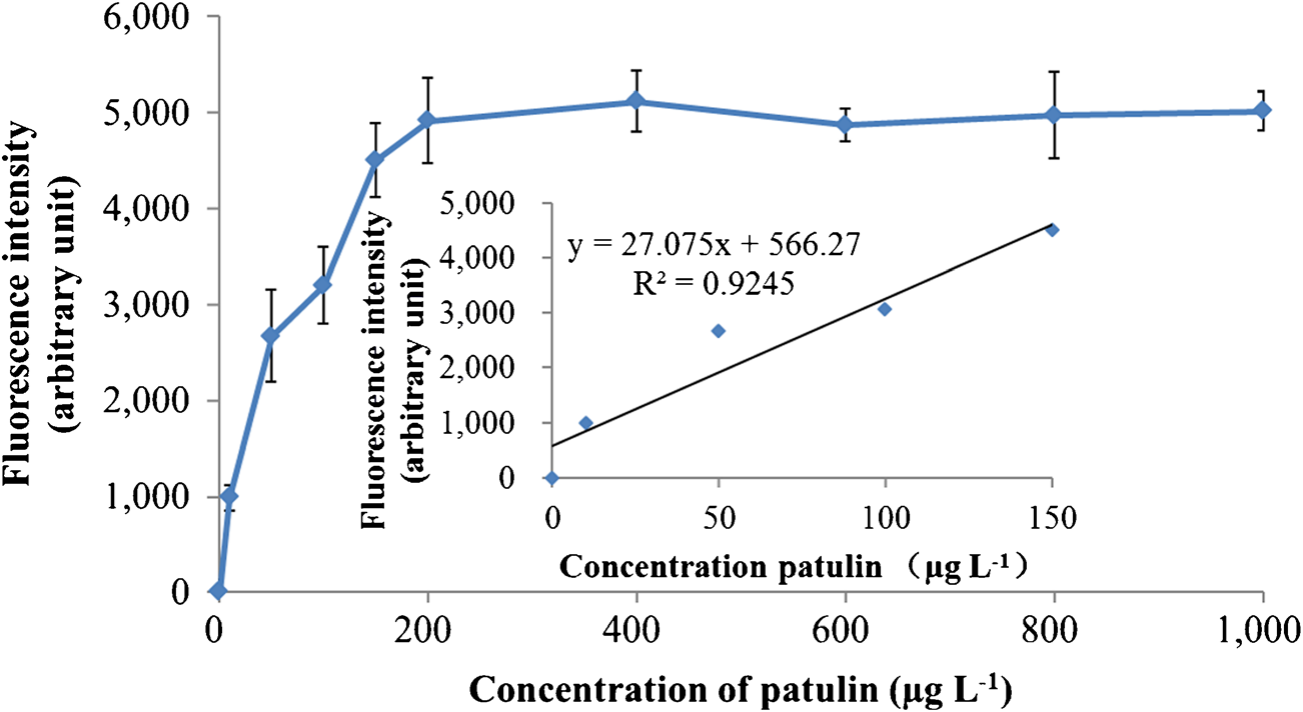
Detection of patulin in apple juice by using the immunoliposome-based fluorometric patulin assay assay. All the experiments were conducted three times (*n* = 3), and data represent as mean ± standard deviation. The fluorescence intensity was measured at an excitation wavelength of 550 nm and an emission wavelength of 585 nm using a fluorescence detector

Apple juice samples with different patulin concentrations (0–1000 μg L^−1^) were analyzed using the optimized assay. Firstly, 1 mL of each sample was mixed with 20 μL of immunomagnetic nanoparticles, and incubated under orbital shaking at room temperature for 1 h. The immunomagnetic nanoparticle-patulin composites were then separated out using magnetic force and washed two times with 0.01 M PBS containing 0.05% Tween 20. Next, 100 μL of the immunoliposome solution was added and incubated at room temperature in the dark for 1 h. During this time, immunoliposomes bound to the patulin-immunomagnetic nanoparticle composites, and then the immunoliposome-patulin-immunomagnetic nanoparticle composites were separated using magnetic force and washed three times with 0.01 M PBS containing 0.05% Tween 20. To lyse the immunoliposomes bound to the patulin-immunomagnetic nanoparticle composites, 260 μL of 30 mM OG was added, then the aliquot of lysed solution (200 μL) was transferred to a 96-well microtiter plate to measure the fluorescence intensity at an excitation wavelength of 550 nm and emission wavelength of 585 nm. The limit of detection for the immunoliposome-based immunomagnetic nanoparticle assay was found to be 8 μg L^−1^, whilst the assay had a linear correlation for patulin detection between 0 and 150 μg L^−1^ (Fig. 2), indicating that the method is capable of quantitative detection. However, detected concentrations higher than 150 μg L^−1^ were shown as the qualitative results.

Multiple different chromatographic methods have been used to detect patulin, including GC with mass spectrometry, HPLC with ultraviolet spectrometry, fluorescence, or mass spectrometry, and TLC. Pennacchio et al. developed a near-infrared fluorescence assay for the detection of patulin in food [7], using a novel fluorescence polarization approach based on emergent near-infrared fluorescence probes. In this method, the fluorophores were coupled to anti-patulin antibodies, allowing patulin to be directly detected in apple juice without any pretreatment [7]. The limit of detection of this method was 0.6 μg L^−1^, lower than the maximum patulin residue limit of 50 μg L^−1^ in European Union regulations [7]. Soldatkina et al. also developed a conductometric enzyme biosensor for patulin, which used a differential pair of gold interdigitated electrodes placed on a substrate for conductometric signal transduction [29]. To prepare a bio-selective membrane, the authors co-immobilized urease with BSA by cross-linking with glutaraldehyde on the transducer surface. The conductometric enzyme biosensor had a linear range of 1 to 50 μg L^−1^; however, higher concentrations of patulin were not assessed. Funari et al. reported a functionalized quartz crystal microbalance sensor using the photonic immobilization technique, which had a detection limit of 140 μg L^−1^ [30]. In this method, the antibody was immobilized on the gold surface of a quartz-crystal microbalance, and a ‘sandwich protocol’, which uses free antibodies in solution as secondary antibodies, was used to detect low-molecular-weight patulin. Similarly, Fang et al. developed a quartz-crystal microbalance sensor based on a molecularly imprinted sol-gel polymer to detect patulin in foods, with a detection limit of 3.1 μg L^−1^ [2]. Compared with the typical sandwich ELISA protocol, the microbalance method had a higher sensitivity and was more rapid, flexible, and portable; however, these methods still require organic solvents to extract patulin. Comparatively, our method does not require organic solvent extraction, detects patulin with good sensitivity and effectiveness, thereby allowing numerous samples to be quantitatively and qualitatively analyzed in a short period.

### Specificity of the assay

**Fig. 3:**
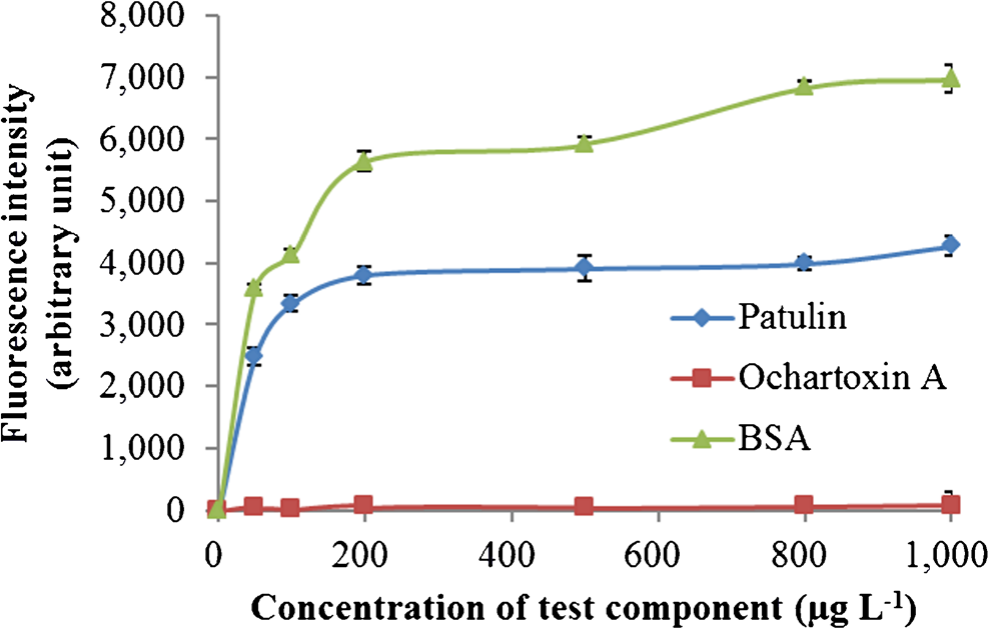
Cross-reactivity of immunoliposome-based fluorometric patulin assay with ochratoxin A and BSA. All the experiments were conducted three times (n = 3), and data represent as mean ± standard deviation. The fluorescence intensity was measured at an excitation wavelength of 550 nm and an emission wavelength of 585 nm using a fluorescence detector

In order to confirm the specificity of the immunoliposome-based immunomagnetic nanoparticle assay for patulin, cross-reactivity was investigated using the mycotoxin, ochartoxin A. As the concentration of ochartoxin A increased from 0 μg L^−1^ to 1000 μg L^−1^, the fluorescent signal remained constant at a level similar to the background signal (Fig. 3), indicating that the assay had no cross-reactivity with ochratoxin A. Conversely, the fluorescent signal increased as the concentration of patulin increased, indicating that the assay had high specificity for patulin. The assay also showed a response to BSA (Fig. 3), indicating cross-reactivity with BSA and potentially limiting its use if BSA is present in food samples.

### Comparison between the fluorometric assay and HPLC

The recovery rates of immunoliposome-based fluorometric assay and HPLC were as shown in Table 2. HPLC analysis revealed that apple juice samples with 0 μg L^−1^, 10 μg L^−1^, 50 μg L^−1^, 100 μg L^−1^, 150 μg L^−1^, 200 μg L^−1^, 300 μg L^−1^, 400 μg L^−1^, and 500 μg L^−1^ patulin had patulin recovery rates of 100 ± 0.00%, 5.82 ± 3.12%, 89.17 ± 0.39%, 93.33 ± 0.70%, 91.11 ± 0.10%, 73.96 ± 0.01%, 94.51 ± 1.0%, 94.83 ± 0.23%and 85.81 ± 0.19%, respectively. The recovery rates of immunoliposome-based fluorometric assay from 0 μg L^−1^ to 150 μg L^−1^ were 100 ± 0.00%, 94.67 ± 0.21%, 96.33 ± 1.12%, 93.03 ± 2.65%, and 99.70 ± 0.16%, respectively. These results indicated that immunoliposome-based fluorometric assay is effective for the detection of patulin in apple juice. However, the recovery rates were reduced when the concentration of patulin in apple juice was higher than 200 μg L^−1^; this will cause inaccurate results when the apple juice is contaminated with high concentration of patulin (Table 2). According to the AOAC method, pre-treating clear apple juice samples involves repeatedly extracting with ethyl acetate for three times, washing with 0.5% sodium carbonate, dehydrating with anhydrous sodium sulfate, evaporating, and redissolving (Fig. 1). At a low patulin concentration (10 μg L^−1^), the patulin recovery rate was very low compared to higher concentrations, suggesting that HPLC may be limited by sample loss during the extensive extraction processes. Furthermore, these processes use organic solvents, such as ethyl acetate, methanol, and acetonitrile, which are toxic to the environment [31]. Unlike HPLC, the immunoliposome-based immunomagnetic nanoparticle assay utilizes IgG-coupled magnetic nanoparticles to separate and concentrate patulin, which is a more specific, highly efficient, and time-saving method. This comparison suggests that the immunoliposome-based immunomagnetic nanoparticle assay is a rapid, sensitive, and environment-friendly method for detecting patulin.

**Table 2:** A comparison of detecting patulin by using HPLC and immunoliposome-based fluorometric assay

Spiked patulin concentration in appile juice (μg L^−1^)	HPLC		Immunoliposome-based fluorometric assay	
	Detected concentration (μg L^−1^)	Recovery rate (%)	Detected concentration (μg L^−1^)	Recovery rate (%)
0 (control)	0 ± 0.00	100 ± 0.00	0 ± 0	100 ± 0.00
10	0.58 ± 0.03	5.82 ± 3.12	47.33 ± 0.00	94.67 ± 0.21
50	44.58 ± 0.19	89.17 ± 0.39	48.16 ± 0.01	96.33 ± 1.12
100	93.33 ± 0.67	93.33 ± 0.70	93.03 ± 0.27	93.03 ± 2.65
150	136.67 ± 0.17	91.11 ± 0.10	149.70 ± 0.24	99.70 ± 0.16
200	147.92 ± 0.02	73.96 ± 0.01	174.16 ± 0.38	87.08 ± 0.18
300	283.63 ± 3.24	94.51 ± 1.0	207.49 ± 4.87	69.16 ± 2.35
400	379.35 ± 0.89	94.83 ± 0.23	273.40 ± 2.72	68.35 ± 0.99
500	428.06 ± 1.10	85.81 ± 0.19	302.81 ± 3.99	60.56 ± 1.31

All experiments were conducted three times, and data represent mean ± standard deviation.

## Conclusions

In order to develop a rapid and sensitive method for the detection of the fungal metabolite mycotoxin, patulin, we developed rabbit anti-patulin-BSA IgG using patulin-BSA conjugates. These antibodies were then used to produce immunoliposomes and immunomagnetic nanoparticles for an immunoliposome-based immunomagnetic nanoparticle assay to detect patulin in apple juice. The assay was able to detect patulin at a concentration of 8 μg L^−1^ in apple juice without the need for extraction, separation, evaporation, or purification. The assay works well for highly contaminated samples and is more environment-friendly than existing methods, which require the use of organic solvents to extract patulin. Therefore, it can be concluded that the assay is a rapid, sensitive, and reliable method for detecting patulin in apple juice and has great potential in a variety of matrices.

## Electronic supplementary material

ESM 1 (DOCX 554 kb)
