# Supplementary material for: Immunoliposome-based fluorometric patulin assay by using immunomagnetic nanoparticles
Source: Mikrochim Acta. 2019 Nov 22;186(12):834. doi: 10.1007/s00604-019-3973-9 (PMC6874700; doi:10.1007/s00604-019-3973-9)
Supplement: Supplementary file 1 — (DOCX 554 kb) [file 604_2019_3973_MOESM1_ESM.docx]

**Electronic Supporting Material on the Microchimica Acta publication entitled**

**Immunoliposome-based fluorometric patulin assay by using immunomagnetic nanoparticles**

**Xinjie Song, Danhua Wang, and Myunghee Kim ***

Department of Food Science and Technology, Yeungnam University, Gyeongsan-si, Gyeongsangbuk-do 38541, Republic of Korea

***Corresponding author,** E-mail: [foodtech@ynu.ac.kr](mailto:foodtech@ynu.ac.kr) (M. Kim)

***Methods***

**IgG purification**

Briefly, antiserum (0.5 mL) was added to two volumes of 60 mM sodium acetate buffer (pH 4.0), gently mixed with 55 µL of caprylic acid for 30 min and kept at 4 °C for 1 h, then centrifuged at 10,000 × *g* and 4 °C for 30 min. The supernatant was collected and filtered with glass wool, added to the same volume of cold-saturated ammonium sulfate, and shaken gently for 30 min. Following centrifugation at 10,000 × *g* and 4 °C for 30 min, the antibody pellet was collected and dissolved in 0.02 M Tris buffered saline (TBS; pH 7.0) containing 1.5 M NaCl and 0.1 % NaN_3_, then dialyzed overnight using a dialysis membrane in 0.02 M TBS.

**Liposome and immunoliposome preparation**

Briefly, DPPE-ATA was prepared by dissolving DPPE (7.2 µmol) and SATA (14.3 µmol) in 1 mL of 0.7% triethylamine in chloroform (v/v). A lipid mixture containing DPPC (40.3 µmol), DPPG (4.2 µmol), and cholesterol (40.9 µmol) was dissolved in 3 mL of chloroform and 0.5 mL of methanol by sonicating for 1 min at 45 °C while flushing with nitrogen gas. Next, 2 mL of encapsulant (100 mM SRB in 0.02 M HEPES buffer; pH 7.5) was immediately added to the lipid mixture and sonicated for 3 min at 45 °C while flushing with nitrogen gas. The organic solvent was then removed by evaporation at 45 °C, leaving a dark purple, gel-like suspension. An additional 2 mL of the encapsulant was added to the lipid suspension and sonicated for 1 min. The mixture was repeatedly vortexed, evaporated, and sonicated until a uniform suspension was obtained, which was sequentially extruded through 0.8 µm and 0.4 µm membrane filters to produce liposomes with a uniform size. The SRB-encapsulated liposomes were dialyzed overnight using a dialysis membrane in 0.01 M HEPES buffer containing 0.2 M NaCl and 0.01% NaN3 (pH 7.5), and then kept at 4 °C in the dark until further use.

Before making the IgG-tagged liposomes, 1 of mg purified rabbit anti-patulin-BSA IgG was dissolved in 1 mL of 0.05 M phosphate buffered saline (PBS) containing 1 mM EDTA and 0.01% NaN3 (pH 7.8). Thereafter, sulfo-KMUS solution prepared by dissolving 2 mg of sulfo-KMUS in 0.1 mL of a solvent mixture of DMSO:methanol (2:1, v/v) was added to 1 mL of the anti-patulin-BSA IgG solution (1 mg mL^-1^) and incubated on an orbital shaker (70 rpm) at room temperature for 3 h. The IgG derivatized with maleimide groups was dialyzed using a dialysis membrane in 0.02 M HEPES containing 0.15 M NaCl and 0.01% NaN3 (pH 7.0) overnight at 4 °C in the dark. In order to deprotect the acetylthioacetate groups on the liposome further, 30 µL of 0.5 M hydroxylamine hydrochloride dissolved in 0.1 M HEPES containing 25 mM EDTA (pH 7.5) was mixed with 300 µL of the liposome solution. The mixture was flushed with nitrogen gas for 1 min and gently shaken at room temperature for 2 h to complete the deacetylation. The pH of the SH-containing liposome solution was adjusted to 7.0 with 0.5 M HEPES solution and mixed with the maleimide-derivatized IgG solution. The mixture was flushed with nitrogen gas for 1 min, incubated at room temperature for 4 h, then incubated overnight at 4 °C in the dark. Next, 100 mM ethylmaleimide dissolved in 0.02 M TBS (pH 7.0) was added to the conjugated liposomes and gently shaken (70 rpm) for 30 min at room temperature to quench the unreacted sulfhydryl groups.

**Immunomagnetic nanoparticle preparation**

Briefly, 0.2 mL of the magnetic iron oxide nanoparticles (with 30 nm diameter) were mixed with 0.2 mL activation buffer in a 1.5 mL tube. Subsequently, 100 µL solution containing 0.5 mg mL^-1^ EDAC and 0.25 mg mL^-1^ NHS was added into the magnetic iron oxide nanoparticles and incubated at room temperature for 10 min with continuous mixing to activate magnetic iron oxide nanoparticles. Thereafter, 0.5 mL of coupling buffer and 0.5 mL of anti-patulin-BSA IgG (2.0 mg/mL) were successively added to the activated magnetic iron oxide nanoparticles and reacted at room temperature for 2 h with continuous mixing. After treating it with 10 µL of quenching solution for 10 min, the reactant was transferred into a disposable culture tube (12 × 75 mm). The quenched reactant was gently washed with 3 mL of washing solution. The disposable culture tube was then inserted into the SuperMag Separator TM magnetic separator and kept at 4 °C for 4 h to separate the anti-patulin-BSA IgG conjugated magnetic iron oxide nanoparticles (immunomagnetic nanoparticles), and then, the buffer was carefully removed to complete the washing.

**Optimization of the assay**

The detection process relies on washing steps to remove unreacted components. First washing (wash I) was performed after the reaction of patulin with immunomagnetic nanoparticles, and second washing (wash II) was performed after the reaction of patulin-immunomagnetic nanoparticle complex with immunoliposomes. To optimize the conditions of the assay, washing frequency of both the washing steps, wash I and wash II, were tested.

***Results and discussion***

**Antibody characterization**

To produce antibodies against patulin, it was necessary to conjugate patulin with a carrier protein because patulin is not immunogenic during antibody development by itself [1, 2]. Therefore, the patulin-BSA immunogen was used for immunization to develop a polyclonal antibody against patulin. Fig. S1 shows the titer of the anti-serum collected from the patulin-BSA immunized rabbit and the titer of the serum collected from the non-immunized rabbit as control. The anti-serum collected from the patulin-BSA-immunized rabbits had a very high titer against patulin-BSA at various dilutions; however, the serum collected from the control rabbits did not. As shown in Fig. S2, the purified anti-patulin-BSA IgG (Lanes 4, 5, and 6) exhibited very high purity compared to the commercial rabbit IgG (Lanes 1 and 2). The antibody (Lanes 4, 5, and 6) and the commercial rabbit IgG (Lanes 1 and 2) showed strong and light bands at around 51 and 25 kDa, respectively. These results confirm that the purity of our rabbit anti-patulin-BSA IgG was comparable to that of commercial rabbit IgG. To investigate the interaction of anti-patulin-BSA IgG with ovalbumin and milk powder, a comparative analysis was performed as shown in Fig. S3. The anti-patulin-BSA IgG has no reaction with ovalbumin and has a slight reaction with skim milk when having a very strong titer against BSA. These results indicated that the anti-patulin-BSA serum may be used to develop an immunoassay for patulin detection.

**Optimization of the assay for patulin detection**

Immunoliposome dilution rate and wash frequency were the most important factors used to assess the assay, as indicated by the fluorescence intensity signals. Excessive washing can result in the loss of immunomagnetic nanoparticles and thus a low fluorescence intensity signal; however, inadequate washing leads to high background signal [3]. To optimize the patulin-detection assay, we analyzed the immunoliposome dilution rate and washing frequency (Fig. S4). When the immunoliposomes were diluted 10-fold with 0.01 M HEPES, the fluorescence intensity signals were beyond the limit of detection due to the high concentration of SRB released by the immunoliposomes (data not shown). Therefore, we chose to dilute the liposomes 20-fold in this assay. Fewer washes (A) during wash II resulted in a higher deviation than more washes (B), even though the fluorescence intensity was higher for A at higher patulin concentrations. Two washes during wash I resulted in the removal of the majority of the un-reacted immunomagnetic nanoparticles from the reaction system. Additional washes (C-E) during wash I resulted in a higher stability and more reliable fluorescence intensity signals than a single wash (A-B). These results were consistent with those of Zhao et al., who developed a rapid immunomagnetic nanoparticle-based immunoassay for the sensitive detection of zearalenone [3]. Our results indicated that three washes after the binding of the immunomagnetic nanoparticles to the immunoliposomes were sufficient to remove any unreacted particles and resulted in a more stable signal. Therefore, two washes after the immunomagnetic bead reaction and three washes after the immunoliposome reaction were selected for the method.

**Liposome, immunoliposome, and immunomagnetic nanoparticle characterization**

SRB-encapsulated liposome particles were prepared using reversed-phase evaporation. Characteristics of the liposomes and anti-patulin-BSA IgG-coated immunoliposomes are shown in Table S1. The average diameter of the liposomes was 196.20 ± 0.66 nm, increasing to 231.50 ± 1.25 nm when the surface of the liposome was coated with anti-patulin-BSA IgG, indicating that the liposome was successfully coated with antibodies to produce immunoliposomes. The inner volume of the liposomes was 3.51 × 10^-12^ µL, with 3.51 × 10^-13^ µmol of SRB encapsulated in the liposome, assuming that the initial SRB concentration was 100 mM. Similarly, Song et al. reported SRB-encapsulated liposome particles containing 5.02 × 10^-13^ µmol of SRB with a size of 229 nm [4].

The polydispersity indices of the liposomes and immunoliposomes were 0.152 ± 0.004 and 0.137 ± 0.008, respectively. During extrusion, the liposome was filtered through polycarbonate filters with pore sizes of 0.4 µm and 0.8 µm, resulting in particles with a homogeneous diameter. A low polydispersity index indicates monodispersed nanoparticles with good stability [5, 6], whilst the potential stability of a colloidal system is indicated by the zeta potential of a liposome solution [7]. The particles in a suspension tend to repel each other when they have a large negative or positive zeta potential, making the suspension stable [8]. As shown in Table S1, both the liposomes and immunoliposomes had negative zeta potentials, indicating that the particles tended to repel each other [9]. Overall, the results indicate that the liposomes and immunoliposomes were stable and homogeneous. They were used to develop a rapid patulin detection method.

The magnetic nanoparticles had a diameter of 30 nm. Rabbit anti-patulin-BSA IgG was used to coat the nanoparticles according to the manufacturer’s instructions. Characteristics of the magnetic and immunomagnetic nanoparticles are listed in Table S1. The magnetic and immunomagnetic nanoparticles had diameters, polydispersity indices, and zeta potentials of 69.71 ± 0.29 nm, 0.329 ± 0.003, and -50.07 ± 2.16 mV; and 128.50 ± 0.93 nm, 0.388 ± 0.013, and -38.80 ± 1.95 mV, respectively. The large size of the magnetic nanoparticles is likely due to the hydrated layer on the surface of the nanoparticle when in the buffer [7, 10]. The size of the immunomagnetic nanoparticles increased to 128.50 ± 0.93 nm following antibody conjugation, indicating that the magnetic nanoparticles were successfully coated with the rabbit anti-patulin-BSA IgGs.

***References***

1. AOAC International (2000) Patulin in Clear and Cloudy Apple Juices and Apple Puree AOAC Official Method 49.7.03
2. Sheu F, Lee O, Shyu YT (1999) The synthesis of antigens and the production of antibodies against patulin derivatives. J Food Drug Anal 7(1): 65-72
3. Zhao F, Shen Q, Wang H, Han X, Yang Z (2017) Development of a rapid magnetic bead-based immunoassay for sensitive detection of zearalenone. Food Control 79: 227-233
4. Song X, Shukla S, Lee G, Kim M (2016) Immunochromatographic strip assay for detection of *Cronobacter sakazakii* in pure culture. J Microbiol Biotechnol 26(11): 1855-1862
5. Urusov A, Zherdev AV, Dzantiev BB (2010) Immunochemical methods of mycotoxin analysis (review). Appl Biochem Microbiol 46: 253-266
6. Bihari P, Vippola M, Schultes S, Praetner M, Khandoga AG, Reichel CA, Coester C, Tuomi T, Rehberg M, Krombach F et al. (2008) Optimized dispersion of nanoparticles for biological in vitro and in vivo studies. Part Fibre Toxicol 5: 1-14
7. Shukla S, Lee G, Song X, Park S, Kim M (2016) Immunoliposome-based immunomagnetic concentration and separation assay for rapid detection of *Cronobacter sakazakii*. Biosens Bioelectron 77: 986-994
8. Karn PR, Cho W, Park HJ, Park JS, Hwang JS (2013) Characterization and stability studies of a novel liposomal cyclosporin A prepared using the supercritical fluid method: comparison with the modified conventional Bangham method. Int J Nanomed 8: 365-377
9. Heurtault B, Saulnier P, Pech B, Proust JE, Benoit JP (2003) Physico-chemical stability of colloidal lipid particles. Biomaterials 24: 4283-4300
10. Chen Y, Wang K, Liu Z, Cui D, He J (2016) Rapid detection and quantification of tumor marker carbohydrate antigen 72-4 (CA72-4) using a superparamagnetic immunochromatographic strip. Anal Bioanal Chem 408: 2319-2327


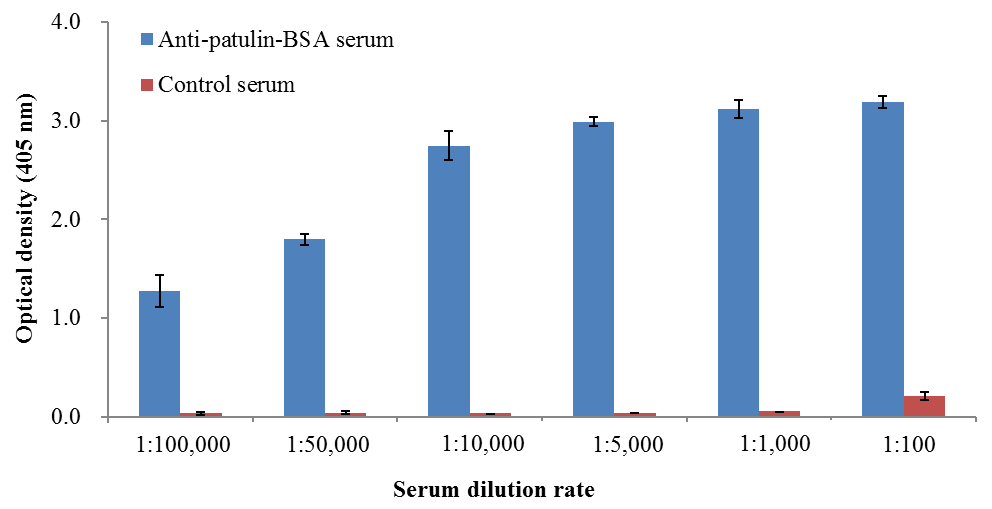


**Fig. S1** Antibody titer of final bleeding serum at different dilution rate

All the experiments were conducted three times (n=3), and data represent as mean ± standard deviation.


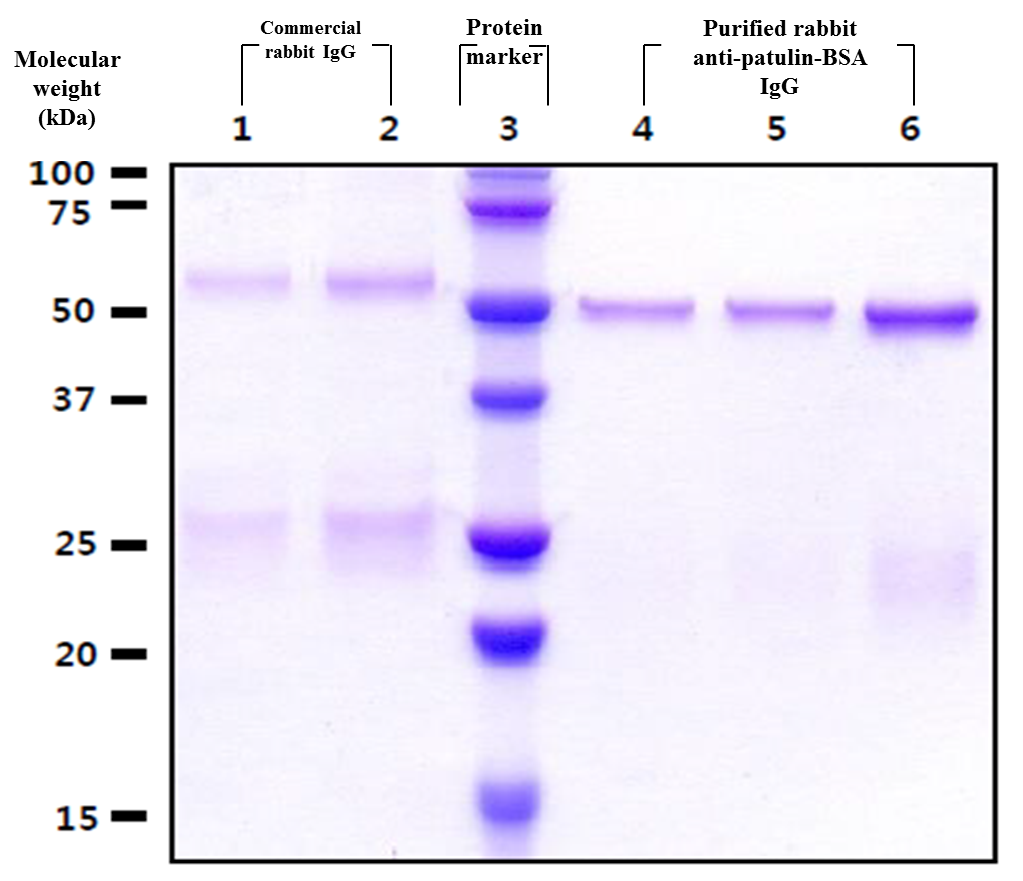


**Fig. S2** SDS-PAGE of developed rabbit anti-patulin-BSA IgG

Lane 1 and 2: commercial rabbit IgG; lane 3: protein marker; lane 4, 5, and 6: purified rabbit anti-patulin-BSA.


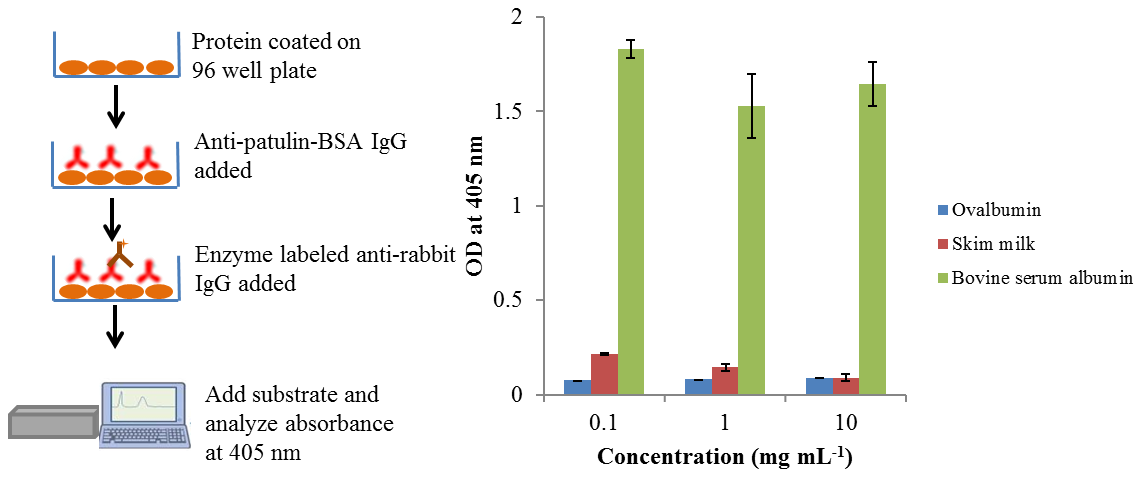


**Fig. S3** Interaction of developed antibody with ovalbumin, skim milk, and bovine serum albumin

The titers were checked by direct ELISA.

All the experiments were conducted three times (n=3), and data represent as mean ± standard deviation.


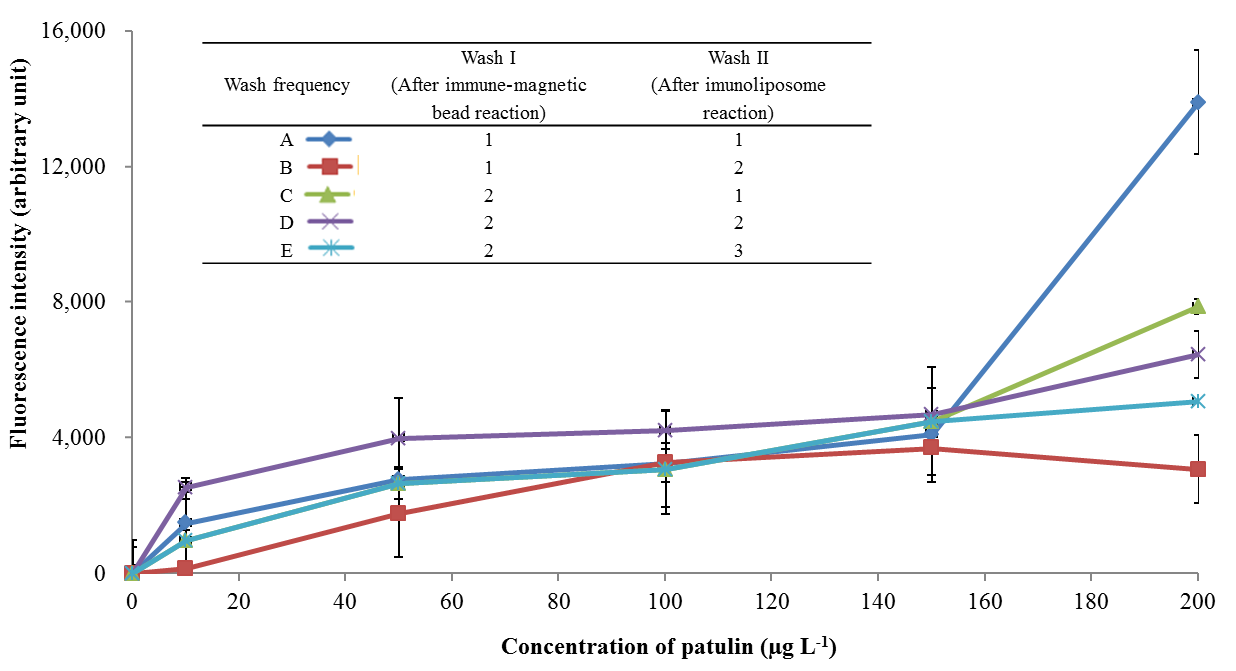


**Fig. S4** Optimization of developed immunoliposome-based immunomagnetic nanoparticle assay

All the experiments were conducted three times (n=3), and data represent as mean ± standard deviation.

The fluorescence intensity was measured at an excitation wavelength of 550 nm and an emission wavelength of 585 nm using a fluorescence detector.


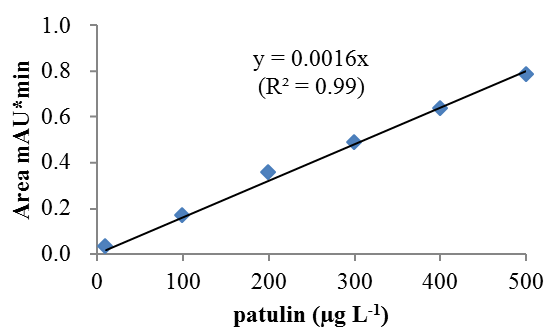


**Fig. S5** Standard curve of HPLC for patulin detection by using HPLC. The patulin was measured on an UltiMate 3000 HPLC at 276 nm

All the experiments were conducted three times (n=3), and data represent as mean ± standard deviation.

**Table S1** Characterization of liposomes, immunoliposomes, and immunomagnetic nanoparticles

|  | Size (nm) | Polydispersity  index | Zeta potential\(mV) | Inner volume  (µL) | SRB µmol/liposome |
| --- | --- | --- | --- | --- | --- |
| Liposomes | 196.20 ± 0.66 | 0.152 ± 0.004 | -27.32 ± 0.64 | 3.51 × 10^-12^ | 3.51 × 10^-13^ |
| Immunoliposomes | 231.50 ± 1.25 | 0.137 ± 0.008 | -17.71 ± 0.46 |  |  |
| Magnetic nanoparticles | 69.71 ± 0.29 | 0.329 ± 0.003 | -50.07 ± 2.16 |  |  |
| Immunomagnetic  nanoparticles | 128.50 ± 0.93 | 0.388 ± 0.013 | -38.80 ± 1.95 |  |  |

All the experiments were conducted three times (n=3), and data represent as mean ± standard deviation.
